# Sequential organization of birdsong: relationships with individual quality and fitness

**DOI:** 10.1093/beheco/araa104

**Published:** 2020-10-30

**Authors:** Sándor Zsebők, Gábor Herczeg, Miklós Laczi, Gergely Nagy, Éva Vaskuti, Rita Hargitai, Gergely Hegyi, Márton Herényi, Gábor Markó, Balázs Rosivall, Eszter Szász, Eszter Szöllősi, János Török, László Zsolt Garamszegi

**Affiliations:** 1 Behavioural Ecology Group, Department of Systematic Zoology and Ecology, ELTE Eötvös Loránd University, Pázmány Péter sétány 1/C, Budapest, Hungary; 2 Centre for Ecological Research, Institute of Ecology and Botany, Alkotmány u. 2–4, Vácrátót, Hungary; 3 The Barn Owl Foundation, Temesvári út 8., Orosztony, Hungary; 4 Department of Zoology and Animal Ecology, Szent István University, Páter Károly u. 1.,Gödöllő, Hungary; 5 Department of Plant Pathology, Szent István University, Villányi út 29–43, HBudapest, Hungary; 6 Ecology Research Group of the Hungarian Academy of Sciences, Pázmány Péter sétány 1/C,, Budapest, Hungary; 7 Theoretical Biology and Evolutionary Ecology Research Group, Department of Plant Systematics, Ecology and Theoretical Biology, ELTE Eötvös Loránd University, Pázmány Péter sétány 1/C, Budapest, Hungary

**Keywords:** fitness, male quality, network analysis, repeatability, syllable sequence

## Abstract

Many vocalizing animals produce the discrete elements of their acoustic signals in a specific sequential order, but we know little about the biological relevance of this ordering. For that, we must characterize the degree by which individuals differ in how they organize their signals sequentially and relate these differences to variation in quality and fitness. In this study, we fulfilled these tasks in male collared flycatchers (*Ficedula albicollis*). We characterized the sequential order of syllables with a network analysis approach and studied the consistency of network variables on distinct time scales (within day, between days, and between years), and assessed their relationship with such quality indicators like age, body condition, arrival date, and fitness related proxies like survival to the next year and pairing success. We found that the syllables were associated nonrandomly with one another and both the frequency differences of consecutive syllables and the number of motif types were higher in the original than in randomized syllable sequences. Average degree and small-worldness showed considerable among-individual differences and decreasing repeatability with increasing time scale. Furthermore, we found relationships between male age and average degree among and within individuals. Accordingly, older males produce syllable sequences by using common syllables less often than younger individuals. However, the network variables showed no relationship with fitness-related variables. In conclusion, the sequential organization of birdsong has the potential to encode individual-specific characteristics, which thus could be used as signal in social interactions and thus potentially could be subject to sexual selection.

## Teaser

The sequential organization of song elements within the songs of the collared flycatcher can potentially function as a reliable signal and provide information about the singing male as it is consistent within individuals and correlates with male age.

## INTRODUCTION

Information about species identity, individual quality, or intention is encoded in diverse ways in signals used in the channels of animal communication (Searcy and Nowicki 2005). One of the general strategies is to code information in the sequences of discrete elements, such as syllables or songs. In simpler cases, such as in insects, acoustic signals contain the repetition of species-specific fixed patterns (Hedwig 2014). In more complex cases, discrete elements can be ordered differently that makes it possible to encode information in various ways possible. We find sequences that can encode diverse, individual- and context-specific information in many species of birds and mammals (Catchpole and Slater 2008; Schlenker et al. 2016). However, we still know little about the evolution of acoustic sequences and how sexual selection acts on these.

One of the best-studied acoustic communication systems is birdsong, which plays a role in territoriality and mate choice (Catchpole and Slater 2008). Birdsong can be regarded as hierarchically organized sequential information (Thompson et al. 1994; Berwick et al. 2011). The smallest repeated units are commonly the syllables that can form repeated sequences within a song, which are called motifs or phrases (Lampe and Espmark 1994; Briefer et al. 2010; Samotskaya et al. 2016). Moreover, songs can add up to various song sequences with a special order of songs types (Slater and Gil 2000; Weiss et al. 2014; Ivanitskii et al. 2016). The particular sequences of discrete elements can differ in the rate of repetition, diversity, combination, and order of syllables (Kershenbaum et al. 2016). This study focuses on the ordering of syllables, which we call sequential organization throughout this paper. We can find large interspecific differences—even between closely related species—in the sequential organization on syllable and song sequence levels (Catchpole and Slater 2008; Lachlan et al. 2010), indicating diverse evolutionary forces that have shaped them even in closely related species (Ivanitskii et al. 2011). Some species use song types, where the order of the syllables is fixed, and different song types follow each other with immediate changes like in the black-headed grosbeak (*Pheucticus melanocephalus*, Bermúdez-Cuamatzin et al. 2018), eventual changes like in the southern house wren (*Troglodytes aedon chilensis*, dos Santos et al. 2015), or with both manners depending on the context like in the genus *Setophaga* (Kaluthota et al. 2019). In other species, the syllables are ordered in diverse ways while not forming song types like in the pied (*Ficedula hypoleuca,*Espmark and Lampe 1993) and the collared flycatcher (*Ficedula albicollis*, Garamszegi et al. 2012) or in the sedge warbler (*Acrocephalus schoenobaenus*, Catchpole 1976).

Not only species but also conspecific individuals can differ in how they organize their song elements sequentially, as it has been shown in common nightingales (*Luscinia megarhynchos*) at the song sequence level (Kipper et al. 2004) and in Cassin’s vireo (*Vireo cassinii*) at phrase sequence level (where phrases are defined as the smallest repeated discrete units separated by 1–2 s long pauses in this species, Arriaga et al. 2016). Such among-individual differences can reflect a neutral variation, but also can be linked to individual quality, whereby the sequential organization of song elements can fulfill signaling functions. The sequential rules at the song sequence level may reflect the age of the males as it was seen in common nightingales (Weiss et al. 2014). Since open-ended learner species are able to modify their syllable repertoire throughout life (Beecher and Brenowitz 2005), the sequential organization of syllables can also change with age. In addition, it is also known that physical condition can affect several aspects of a birdsong, such as tempo (Nishida and Takagi 2018), frequency (Galeotti et al. 1997), amplitude (Ritschard and Brumm 2012), and song rate (Yamada and Soma 2016), which may also interfere with how individuals organize their songs sequentially. As different syllable combinations may incur different production costs (Podos 1997, Geberzahn and Aubin 2014), we can also hypothesize that the physiological condition of individuals can influence the sequential organization of syllables, which can also translate into differences in fitness. In this study, we focus on revealing the relationships between the sequential organization of syllables and the quality and fitness of the singing male.

Our model species, the collared flycatcher, is an intensively studied insectivorous, migratory, hole-nesting passerine with breeding ranges mostly in Central and Eastern Europe. In the courtship period, males defend their territory and attract females with their songs. The songs are 3–5 s long acoustic sequences consisting of different syllables. Individuals typically express a moderately large repertoire of approximately 45 syllable types in 20 songs sampled (Garamszegi et al. 2012; Zsebők et al. 2017; Zsebők, Herczeg, et al. 2018). Previously we showed that the quantitative and qualitative features of song (such as song rate, duration, frequency attributes, and repertoire content) can indicate different aspects of individual quality and that these traits can potentially mediate female choice (Garamszegi et al. 2004, 2007, 2008, 2012; Garamszegi, Merino, et al. 2006; Hegyi et al. 2010). However, the sequential organization of syllables within songs and its relationship with individual quality has not been studied so far in this species.

**Figure 1:**
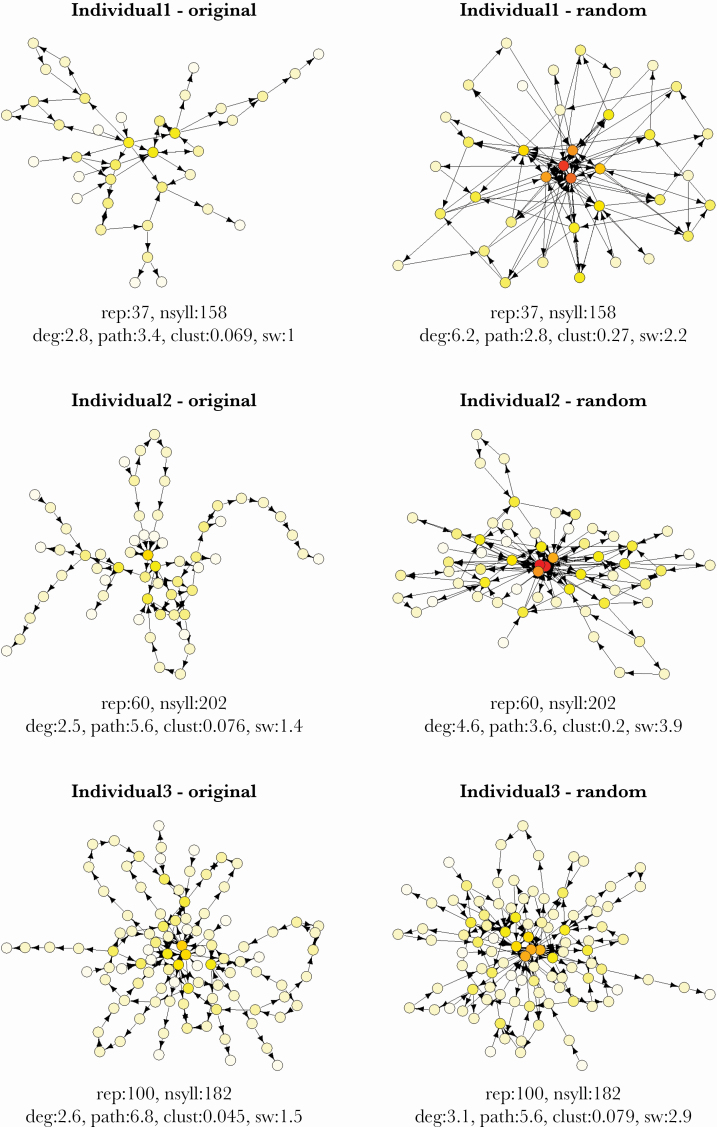
Original and random syllable sequences of three collared flycatcher males shown as networks. On each plot, the ID of the individual is indicated in the title; the derived network variables are shown below the graph (rep: repertoire size, nsyll: number of syllables, degree: average degree, path: average shortest path, clust: clustering coefficient, sw: small-worldness). Circles represent particular syllable types; their colors indicate the degree (higher degrees are darker). The arrows between circles indicate an incidence that the particular syllable types sung after each other within the songs.

One straightforward method to characterize the patterns of the sequences of acoustic elements is network analysis. When using this approach, the different elements of the sequence are the nodes, and the connections between them represent the adjacency of the elements in the sequence; in other words, it describes the first-order relationship between the elements (Kershenbaum et al. 2016). In directed networks, the connections have directions and thereby indicate the syllable order in the sequence, as they naturally follow each other in the song performance (see Figure 1 for some examples). Generally, the graphical representation of the network may be applied to describe the overall sequence organization of songs and to form biologically relevant hypotheses (Roach et al. 2012; Opaev 2016; Benítez Saldívar and Massoni 2017). The application of network approach can provide us with measures that can be used to statistically explore the within-individual and the among-individual variation in the sequences of syllables. The four most common network variables are average degree, average shortest path, clustering coefficient, and small-worldness (Table 1). The average degree is high when the syllable types can be found adjacent to many other syllable types in the songs. The average shortest path increases with the length of the stable sequences containing unique syllable types. The clustering coefficient is high when several syllable types are frequently adjacent to one another. Lastly, the value of small-worldness is high when most of the syllable types are not adjacent to one another, but most of them are only a small number of steps apart in the network, for instance, if sequences containing mostly unique syllables are connected by some common syllable types.

**Table 1:** Description of variables that characterize the sequential organization of syllables. Two classical song traits (1–2) and four network variables (3–6) are described in the contexts of network theory in general and song organization in particular

Variables	Implication for network structure	Implication for song organization
(1) Number of nodes	The size of the network	Number of syllable types (repertoire size)
(2) Sample size	Number of observations used to describe the associations among units	Number of syllables
(3) Average degree	Average number of the connections between nodes	Average number of instances when syllable types are follow other syllable types within the performance of songs
(4) Average shortest path	Average measure of the shortest paths between the nodes	Describes how far the syllable types follow each other in a sequence within a song on average
(5) Clustering coefficient	A global measure of the number of nodes creating triangles	Describes the relative occurrences of cases when three syllable types neighboring each other in all paired combination
(6) Small-world coefficient	A global measure of such phenomenon when few nodes are connected to each other directly but in the same time any nodes can be reached in small number of steps	Describe the phenomenon when small number of syllable types occur next to each other in the sequence, but in the network they can be reached in few steps

Several studies have described the sequential organization of song elements by using network variables, and suggested that the first-order relationship between the elements can have biological relevance. This method has been successfully used before to characterize species-specific sequential organization in the southern house wren (Deslandes et al. 2014), individual differences in the grey crowned warbler (*Seicercus tephrocephalus*, Opaev 2016) and the California thrasher (*Toxostoma redivivum*, Sasahara et al. 2012; Cody et al. 2015). Moreover, in the common nightingale, the network variables calculated at the song sequence level were related to the context and age of the singing birds (Weiss et al. 2014). On the basis of these studies, network analysis appears to be a reliable approach to characterize the within-individual and among-individual differences in the syllable sequences in our model species.

In this study, we hypothesized that sequential organization of birdsong can potentially function as reliable signal and relate to male quality and fitness. We tested several predictions to investigate these hypotheses. We predicted that the sequential organization is not random, and affected by the acoustic similarity of adjacent syllables and the existence of higher order motifs. We also predicted that individuals consistently differ in how they order their syllables, and these among-individual differences in sequential organization predict among-individual differences in male quality and fitness. To test these predictions, we characterized the sequential organization of syllables with network variables (average degree, average shortest path, clustering coefficient, and small-worldness), and calculated the repeatability of the network variables on three different time scales (within day, between days, and between years). We compared the original and randomized sequences regarding the values of network variables, the spectral frequency of syllables, and the detected higher order motifs in the syllable sequences. We also tested the relationships between the network variables and traits reflecting male quality and fitness. For that, we used three male traits as proxies of male quality: age, body condition, arrival date. Age and arrival date are known quality indicators in collared flycatcher as they affect the pairing success and pairing speed (Szász et al. 2019), and body condition is frequently linked to breeding success and survival in birds (Labocha and Hayes 2012). We used two traits as fitness components: pairing success and survival to the next year of males.

## METHODS

### Field data and acoustic analysis

The sound recordings were made at our field station installed with approximately 800 nest-boxes in Pilis-Visegrádi Mts., Hungary (47° 43′ N 19° 01′ E) in the courtship season (April–May) between 1999 and 2015. We recorded the songs of singing males for at least 5 min (recording a minimum of 20 songs) in the morning hours between 6 and 12 AM with a parabolic microphone (Telinga parabolic dish and Sennheiser ME-62 microphone with a P6 preamplifier) and digital sound recorders (Tascam DR1 or Microtrack II). For the general protocol of the sound recording, see Garamszegi et al. (2012). After recording, we caught the males in their nest-box using spring traps and individually marked them with numbered aluminum rings. Furthermore, using brush pen, we colored their belly feathers with individual color combinations (abraded only in a few weeks) to identify the focal bird after a few days later without further capturing. We measured their weight with Pesola spring balance to the nearest 0.1 g, and tarsus length with caliper to the nearest 0.1 mm. We also determined their minimum age on the basis of their feather colors (Svensson 1984) and our long-term ringing database (ranging back to 1989), then we released them. For the detailed handling protocol, see Garamszegi et al. (2007, 2012) and Garamszegi, Merino, et al. (2006). Later on, we regularly monitored the nest-boxes to follow the birds to record their life history.

We used recordings from 176 individuals, where each individual is represented by one recording. We use this dataset to describe the general characteristics of sequences, to compare the original sequences to random sequences, and to test the relationships with male traits related to quality and fitness.

Additionally, to characterize the consistency of the sequential organization of song, we obtained repeated recordings from individuals within the same day (34 males, time lag between recordings, mean ± SD: 6.3 ± 2.8 min), within the same season but on different days (24 males, time lag between recordings: 2.9 ± 1.6 days), and among different years (13 males, time lag between recordings: 1.3 ± 0.5 years). The time between the recording and the catching was usually not more than an hour. Based on our long-term experience, the owner of the nest-box did not change during such a period of time. However, we did not use data from recordings for which the identity of the individual was ambiguous. In the case of between-day repeated recordings, we identified the birds based on their color marks without capturing again. In the case of between-year repeated recordings, we did not know the identity of the bird in advance in the second year, thus we identified the birds based on their ring number after catching them.

For the acoustic analyses, we used the program Ficedula Toolbox (open-source, freeware, https://github.com/zsebok/Ficedula, Zsebők, Blázi, et al. 2018). We selected the first 20 good quality recordings with a low level of background noise for further analyses. Initially, we cut out the songs from the original recordings and then segmented them into syllables by marking the start- and endpoints, as well as the minimum and maximum frequencies of each syllable. Finally, we categorized the segmented syllables manually into syllable types on the basis of their spectrographic representation. For this last step, for each syllable, the program showed the most similar syllable types that could be interpreted being the same syllable type. From this automatic recommendation, the user is expected to choose a syllable type category, or define a new one if the criterion for the match cannot be approved by human judgment. The pair-wise similarities of the syllables were obtained by calculating the Euclidean distance based on the following five classical acoustic variables: the duration, the minimum, maximum and mean frequency, and the frequency bandwidth of the syllable, where each acoustic variable was standardized to have a range between −1 and 1. This clustering process with the Ficedula Toolbox was proved to be highly repeatable between observers on the collared flycatcher song (Cohen’s *κ* = 0.87, Zsebők, Blázi, et al. 2018). This process resulted in the list of the unique identifiers of syllables in the sequence as they follow one another in the song performance of an individual.

### Generating networks from syllable sequences and obtaining network variables

We obtained the network variables for directed networks. First, we calculated the adjacency matrix (Croft et al. 2008) of the syllable types for each song separately on the basis of the sequences of syllable types. In this matrix, the syllable types found in a given individual were listed in the rows and columns, and the number in the cells reflected the number of the incidences of association as defined on the basis of their ordering within song. Thereafter, we summed up the 20 matrices for the given recording, resulting in a summed adjacency matrix for the whole recording. From each matrix, we created the directed unweighted network, where the nodes represented the syllable types. Note that AB and BA sequences were differentiated and so the resulted adjacency matrix was asymmetric and the obtained unweighted network represented the directed connections between syllable types if there was at least one occasion when they followed each other within a song.

We calculated two classical and four network-based variables to describe the sequential organization within the songs for each recording (see Table 1 for a detailed explanation). The two classical variables were 1) repertoire size (the number of unique syllable types detected in the recording, which is also identical to the number of nodes in the corresponding network of syllables) and 2) number of syllables in the whole recording (underlying the sample size for the network analysis). The network analyses provided us with the following variables: 1) the average degree of the network, 2) the average shortest path, 3) the clustering coefficient, and 4) the small-worldness coefficient. The small-worldness coefficient was calculated according to Humphries and Gurney (2008).

We applied the “igraph” package (Csardi and Nepusz 2006) in the R environment (R Core Team 2019) for the calculation of network variables for directed networks. The R script for the calculation of variables is provided in the Supplementary Material.

### Evaluating differences from random sequence patterns

We tested if the network variables obtained for the original sequences could be discriminated from network variables that corresponded to randomized syllable sequences to see whether the syllables follow one another systematically or randomly. Accordingly, we generated 100 randomized syllable sequences for each recording, where the order of the syllables was randomized within the songs, and obtained the network variables. Subsequently, we calculated Cohen’s *d* effect size (Cohen 1988), reflecting the difference between the network variables obtained from the original sequence and the distribution of 100 measurements obtained on the randomized sequences. We then repeated this process for all recordings and network variables, resulting in 176 effect sizes for each network variable. For each network variable, we calculated the overall effect size by building a meta-analytic model weighted with the variance of the samples by using “metafor” (Viechtbauer 2010) and “MAd” libraries (Del Re and Hoyt 2014) in R.

We also investigated if the spectrographic properties of the detected sequences can be differentiated from that of random sequences, which may suggest some physical constraints behind song organization. Accordingly, based on the acoustic measurements of syllables provided by the Ficedula Toolbox, we calculated the absolute value of differences in mean frequencies between the consecutive syllables in the original sequences for all recordings. We repeated the analysis by using a randomized order of the syllables within the songs. Considering the large number of randomly arranged syllable pairs within recordings, we used only one randomized sequence here. For each recording, we calculated the Cohen’s *d* effect size reflecting the differences between the frequency shift between consecutive syllables obtained from the original and randomized sequences for each recording. Finally, we calculated the overall effect size in the same way as described above.

Furthermore, we studied the number of motif types to reveal whether the nonrandom ordering of syllables can be explained by the existence of repeated syllable sequences. We defined the motif as such repeated syllable sequence that appeared in more than one song in the recording. We used a custom made R script to find the motifs with length of 2–10 syllables. After finding all the motifs in each recordings, we calculated the number of different motif types at each motif length. We also applied these calculations to the same recordings in which we randomized the order of syllables within songs. For each motif length, we tested the difference of number of motif types between the original and randomized sequences with Wilcoxon signed-rank matched-pair test (altogether 10 tests).

### Correcting for repertoire size and sample size

Theoretically, network variables are not independent of one another, and the number of nodes (repertoire size) may also affect the outcome of the network statistics (Sasahara et al. 2012; Weiss et al. 2014; Hedley 2016; Opaev 2016). We initially calculated pair-wise Pearson’s correlation coefficients among all the six variables to explore their relatedness. We adjusted the *P* values on the basis of the number of tests performed (altogether 15 tests) following the Bonferroni method to control for multiple testing. As we found strong correlations between the classic acoustic and network-based variables (see Results), we re-calculated the network variables relative to repertoire size and syllable number. To do so, we built linear models (LMs) in which we included one network variable as the dependent variable and entered repertoire size and syllable number as independent variables to control for their confounding role. We derived the residuals from these models and subsequently used them throughout the manuscript as network variables that are independent of repertoire size and the number of syllables in the recording. To visualize the relationship between the variables, we performed principal component analysis (PCA) and depicted their contribution to the main principal components on graph with the help of R packages “factoextra,” “FactoMiner,” and “shape” (Lê et al. 2008; Soetaert 2018; Kassambara and Mundt 2020). The related statistics are shown in Supplementary Tables S1–S4. Please note that we used PCA only for characterizing the relationships among the variables.

### Repeatability

To describe the consistency in sequential organization, we calculated the repeatabilities of each network variable along the following three different time windows: within-day (34 males), between-day (24 males), and between-year (13 males) scales, in which each individual was represented by two recordings with the same number of songs within individual (in average 19.8 ± 2.1 songs per recording). The repeatability was determined by decomposing the variance of the variables into between-individual (*V*_between-individual_) and within-individual (*V*_within-individual_) variance components by means of linear mixed models (LMMs), where the random factor was the individual ID and the dependent variable was the focal network variable. We also included the age of males in the models as a covariate because previously, it turned out that it had a significant effect on syllable usage (Garamszegi et al. 2012). Finally, we calculated the adjusted repeatability according to the following equation:

$$\begin{array}{l} R=\frac{V_{between-individual}}{V_{between-individual}+\;\;\;V_{within-individual}} \end{array}$$

We determined the confidence interval (CI) around the repeatability estimates by parametric bootstrap with 1000 iterations (Nakagawa and Schielzeth 2010). The estimate of repeatability was considered significantly different from zero if the calculated CI did not include zero. For the detailed description of repeatability and calculations, see our previous work (Zsebők et al. 2017).

### Relationship with male traits related to male quality

We used age, arrival date, and body condition and two network variables (average degree and small-worldness) to investigate the relationship between male quality and the sequential organization of songs. Because we predicted that only those song characteristics can function as signals that have significant repeatability, we did not assess the relationship between male traits and other network variables for which the repeatability was not significantly different from zero at any time scale. Body condition was calculated from the weight and tarsus length of males according to Peig and Green (2009). Age was handled as an integer variable and calculated from the ringing data (the mean age of males was 1.9 ± 1.1 years). Arrival date was estimated as the day of the first occurrence of the focal male in the breeding site that was standardized across years using the population median for the given year as a reference for zero values (Garamszegi et al. 2012). We built LMs separately for each network variable as the dependent variable. In all models, body condition, age, and arrival date were used as continuous fixed effects. We included year as a discrete fixed effect in the models to control for the between-year effect. We tested the significance of the fixed effects with likelihood-ratio tests. Because the distribution of small-worldness deviated largely from a normal distribution, we used logarithmic transformation on this variable.

Given that we found a relationship between age and average shortest path in the among-individual analyses (see Results), we also tested the within-individual changes in these variables with paired *t*-test for the 13 individuals for which we had records from two different years.

### Relationship with fitness components

We calculated the fitness components as follows. For the estimation of survival, we used the recapture history of individuals, and determined if they were also captured in the year after the year of song recording. A previous study found that the males in our collared flycatcher population are highly philopatric (129 m mean distance between nesting localities in consecutive years, Könczey et al. 1992), and more accurate survival estimates provide very similar results to recapture probabilities (Garamszegi 2004). Survival to the next year was handled as a binary variable: Males that were also present in the next year in our breeding site received a score of 1; otherwise, they received a score of 0. Pairing success in the focal year was also handled as a binary variable: Males that established a nest and were found with 8 day-old chicks received a score of 1; otherwise, they received a score of 0. See also Hegyi et al. (2010) and Garamszegi et al. (2012) for additional details for the calculation and use of these fitness-related variables.

Thereafter, we investigated the relationships between the fitness components and the network variables with generalized linear models (GLMs) with binomial distribution. We built models separately for the two fitness variables in which the fitness variables were the binomial dependent variables, and the network variables were the continuous fixed effects. In both models, the age, arrival date, and body condition of males were also included as continuous fixed effects and the year as discrete fixed effect to control their additional effect on the fitness component (Szász et al. 2019). We tested the effect of the fixed effects with likelihood-ratio tests.

For all statistical computations and visualizations, we used the R environment and its libraries “lme4” (Bates et al. 2015), “car” (Fox and Weisberg 2011), and “ggplot2” (Wickham 2009).

## RESULTS

### Descriptive characteristics of sequences

The network approach revealed several important characteristics of syllable sequences in the song of collared flycatchers (Figure 1). The networks frequently contained long arms without branching and in average, 50.3 ± 10.7% of nodes had a degree less than 3 (having one or two connections). This indicated that several syllable types occurred only next to one another in the sequence. Only 13.6 ± 6.4% of the connections had two directions indicating that most of the consecutive syllables followed each other only in one way. 34.5 ± 9.6% of the connections had weight more than 1, showing that considerable amount of syllable pairs occurred repeatedly in the recordings. The network variables largely varied across the individuals, with the highest coefficient of variation (over 50%) for the clustering coefficient (Table 2).

**Table 2:** Descriptive statistics of the classical and network variables (*N* = 176)

	Mean	SD	CV (%)	Minimum	Maximum
Repertoire size	47.0	19.8	42.1	12	105
Number of syllables	194.1	49.4	25.4	99	374
Average degree	3.5	0.7	19.7	2.2	6.0
Average shortest path	4.8	1.5	31.4	1.9	10.6
Clustering coefficient	0.14	0.07	51.5	0.00	0.51
Small-worldness	1.7	0.7	42.4	0.0	5.8

Several network variables strongly and significantly correlated with each other and the repertoire size and number of syllables (Table 3, Supplementary Figure S1A). Therefore, we used the network variables that were controlled for repertoire size and number of syllables. The resulted network variables were also largely correlated to each other, only averaged degree and small-worldness had no significant correlation (Table 3, Supplementary Figure S1B).

**Table 3:** Relationships among the studied classical and network variables (*N* = 176)

	Repertoire size	Number of syllables	Average degree	Average shortest path	Clustering coefficient	Small-worldness
Repertoire size		**0.55**	0.00	0.00	0.00	0.00
Number of syllables	**0.55**		0.00	0.00	0.00	0.00
Average degree	**−0.41**	0.21		−**0.47**	**0.47**	0.21
Average shortest path	**0.82**	**0.42**	−**0.55**		−**0.24**	−**0.43**
Clustering coefficient	**−0.58**	−0.17	**0.61**	−**0.59**		**0.67**
Small-worldness	**0.29**	**0.22**	0.07	0.00	**0.35**	

Pearson’s correlation coefficients are shown for the original variables in the lower triangle, and for the variables after correction for repertoire size and number of syllables in the upper triangle. The significant correlations (*P* < 0.05) are indicated in bold. The *P* values were corrected for multiple testing based on Bonferroni method (original *P* values were multiplied by 15). For the visual representation of these relationships, see Supplementary Figure S1.

### Comparison to random sequences

**Figure 2:**
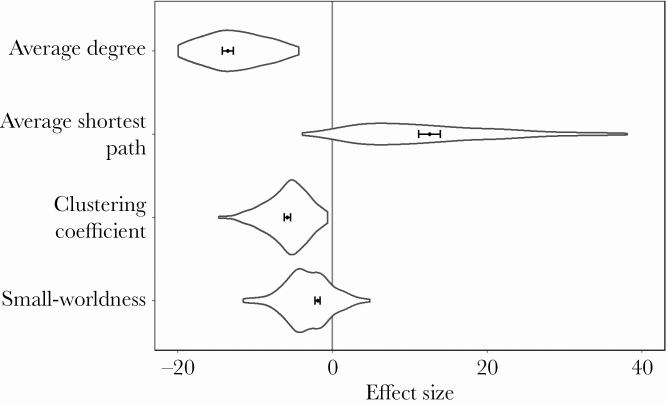
The distribution of the Cohen’s *d* effect sizes for network measures. The effect sizes were calculated based on the comparisons of the estimates that were obtained from the observed data with those that correspond to the randomized sequences of syllables depicting the null distribution of expectancy (*N* = 176 individuals, 100 repeats). The violin plots show the distribution of individual effect sizes. The whisker plots show the CIs of the mean effect sizes calculated via a meta-analytical approach.

We compared the network variables that were estimated from the observed syllable sequences with estimates that corresponded to randomized sequences reflecting the null distribution (Figure 2, Supplementary Table S5, see also Figure 1 for visual comparison of networks). Cohen’s effect sizes depicting individual deviations from the null distribution covered a large range, which had CIs that could be statistically distinguished from zero for all network variables. This generally indicated that the detected syllable order was considerably different from what could be expected by random chance. In particular, for the real data, average shortest path were higher, while average degree, clustering coefficient and small-worldness were smaller than for randomized sequences.

The absolute value of difference between mean frequencies of consecutive syllables was 1913.9 ± 311.5 Hz in the original sequences and 1657.5 ± 229.7 Hz in the random sequences. We found this frequency difference between the original and random sequences significant (the overall effect size for the difference was 0.21 with CI: 0.19–0.23; Supplementary Figure S2).

The study of motif types in the original sequences revealed that most of the individuals used at least 1 motif type up to 5 syllables, and 15.9% of individuals produced at least 1 motif type with 10 syllables. Contrary to that, we found motifs with 5 syllables in less than 0.6% of the cases in the randomized sequences (0 ± 0.1 motif types in average) and no recordings with motifs of 10 syllables. We found that the original sequences contained significantly more motif types than the randomized sequences at all motif length (Table 4). To characterize the relationship between the number of motif types and the average shortest path, additionally, we calculated the correlation between them. We found that they are significantly and positively related (Pearson’s correlation, at motif length 2: *R* = 0.34, *P* < 0.001; at motif length 5: *R* = 0.21, *P* = 0.004; and at motif length 10: *R* = 0.18, *P* = 0.017; df = 174 at all motif length).

**Table 4:** Statistics of the motifs in the original and randomized sequences

	Original sequences			Randomized sequences				
Motif length	Mean ± SD	Min-max	Percent of individuals	Mean ± SD	Min-max	Percent of individuals	W	*P*
2	29.2 ± 10.1	6–69	100.0%	9.4 ± 6.4	0–37	99.4%	15,520	<0.001
3	23.3 ± 9.3	2–61	100.0%	1.3 ± 2.1	0–14	50.0%	15,576	<0.001
4	16.5 ± 8.4	0–49	99.4%	0.1 ± 0.5	0–3	9.1%	15,400	<0.001
5	11 ± 7.5	0–39	96.6%	0 ± 0.1	0–1	0.6%	14,535	<0.001
6	6.9 ± 6.4	0–31	85.8%	0 ± NA	0–0	0%	11,476	<0.001
7	4.1 ± 4.9	0–24	69.9%	0 ± NA	0–0	0%	7626	<0.001
8	2.1 ± 3.3	0–17	52.3%	0 ± NA	0–0	0%	4278	<0.001
9	1 ± 2.1	0–10	30.7%	0 ± NA	0–0	0%	1485	<0.001
10	0.5 ± 1.3	0–8	15.9%	0 ± NA	0–0	0%	406	<0.001

Beside of the mean ± SD, min – max values of the number of motif types, the table shows the percent of individuals that used at least one motif type with the given length. Wilcoxon signed-rank test for matched pairs was applied to test the difference between the motif number of original and randomized sequences (W statistics and *P* values are shown, *N* = 176).

### Repeatabilities

**Figure 3:**
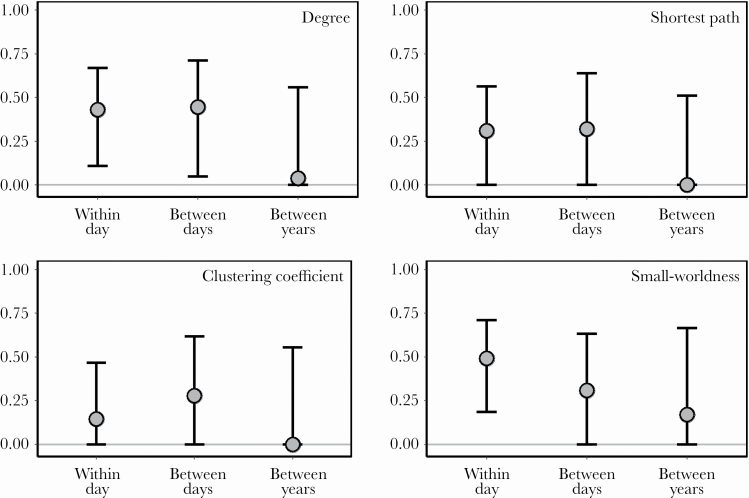
Repeatabilities of the network variables in three temporal windows (within day: *N* = 34; between days: *N* = 24, between years: *N* = 13). The circles show the estimated repeatabilities; the whiskers indicate the bootstrap-based 95% CIs (1000 repeats).

Two out of the four network variables (average degree and small-worldness) had repeatabilities that were significantly different from zero in at least one time scale (Figure 3, Supplementary Table S6). The estimated mean repeatabilities appeared to differ among the variables and time scales, but the corresponding CIs largely overlapped. We found repeatabilities higher than 0.4 in the within-day and between-day contexts for the average degree and in within-day for small-worldness. At the between-year scale, all the repeatabilities were statistically indistinguishable from zero.

### Relationships with male traits

**Figure 4:**
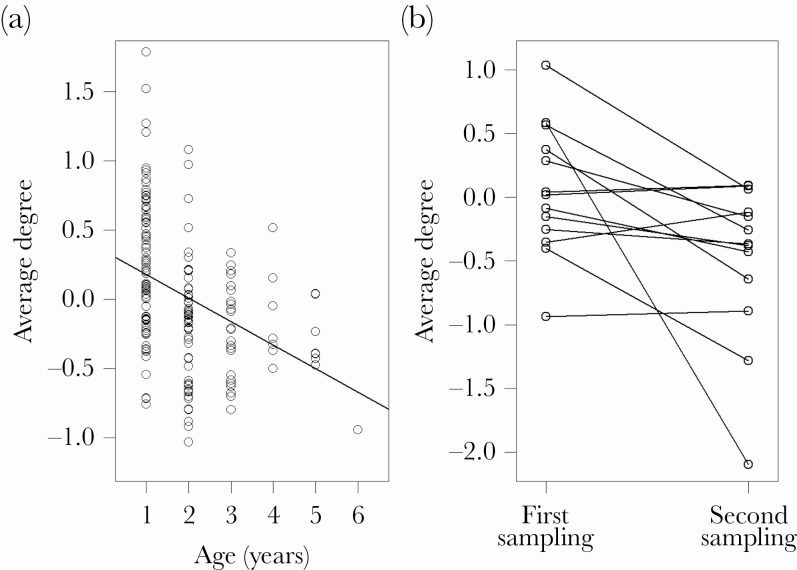
The relationship between the average degree and age of males (A) among individually (*N* = 176) and (B) within individually (*N* = 13). For the underlying statistics, see Results and Table 5.

Male age was significantly and negatively related to the average degree even after the correction of *P* values for multiple testing (Table 5, Figure 4A). We tested whether within-individual changes in the network variables showed systematic patterns to uncover the underlying mechanism that may mediate this age effect in the among-individual analyses. We found a significant within-individual decrease for average degree (paired *t*-test, *t* = −2.0, df = 12, *P* = 0.03, Figure 4B). These results indicate that older individuals compose their sequences with syllable types that are less common in other sequences compared to younger individuals.

**Table 5:** Relationships between the network variables and quality related male traits

	Average degree			Small-worldness		
	T	χ ^2^	*P*	*t*	χ ^2^	*P*
Age	−**4.80**	**23.02**	1.6*10 ^**–6**^	0.03	0.00	0.98
Arrival	1.24	1.55	0.21	0.01	0.00	0.99
Condition	0.93	0.87	0.35	−1.69	2.84	0.09
Year		25.5	0.02		14.43	0.34
	*R* ^2^ = 0.26			*R* ^2^ = 0.10		

One LM was built for each network variable. Likelihood-ratio tests were applied; the *P* values shown here were not corrected for multiple comparison. Relationships remained significant after Bonferroni correction are indicated in bold (*N* = 176, df_year_ = 13, otherwise df = 1).

The arrival date and body condition were not significant predictors of any network variable (Table 5). We also found no significant relationships between the network variables and survival or pairing success (Table 6).

**Table 6:** Relationships between the network variables and the fitness-related variables

	Pairing success			Survival to the next year		
	z	χ ^2^	*P*	z	χ ^2^	*P*
Average degree	0.39	0.15	0.70	−1.36	1.90	0.17
Small-worldness	−0.66	0.43	0.51	1.93	4.06	0.04
Age	0.06	0.00	0.95	−0.43	0.18	0.67
Condition	−0.92	0.89	0.35	2.33	6.27	0.01
Arrival	−2.71	7.87	0.01	0.14	0.02	0.89
Year		48.76	<0.001		22.04	0.06
	*R* ^2^ = 0.27			*R* ^2^ = 0.16		

One GLM was built for each fitness related variable. Likelihood-ratio tests were applied; the *P* values were not corrected for multiple comparisons (*N* = 176, df_year_ = 13, otherwise df = 1).

## DISCUSSION

Our network analysis successfully revealed several aspects of the sequential organization of syllables. We found that the syllables within the songs of the collared flycatcher were ordered nonrandomly, and we showed that the order is influenced by the acoustic features of the syllables and that motifs exist. The consistent between-individual differences are supported by the non-zero repeatability of some network variables, which depict a decreasing tendency as the temporal window for the repeated sampling increases. Furthermore, we found a relationship between male age and average degree at between- and within-individual levels. However, we found no statistical evidence that fitness components were related to the sequential organization.

### Sequential organization of the song

In the songs of the collared flycatcher, the syllables apparently occur in a limited number of sequences: the average degree, clustering coefficient, and small-worldness were smaller, and the average shortest path was higher than it would have been expected from the random sequences of syllables. Several not mutually exclusive physical and cognitive constraints can explain these patterns.

We know that there are some production costs associated with specific combinations of syllables that predict their association. For instance, in the *Emberizidae* family, syllables that have a large frequency bandwidth and are connected with a short pause between them might be challenging to produce (Podos 1997). A similar physical constraint may apply to situations, when a large difference exists between the frequency at the end of a syllable and that of the start of the consecutive syllable (Geberzahn and Aubin 2014). On the other hand, if females prefer males with “sexy sequences” similarly to the sequence of the “sexy syllables” of canaries that are physically difficult to produce (Drăgănoiu et al. 2002), then these attractive sequences are expected to be expressed in the songs frequently. In this study, we revealed that males tend to organize their syllable sequences in a way that the consecutive syllables have larger differences in their mean frequency than by chance; therefore, it partly can be responsible for the sequential organization. Based on the papers cited above, we assume that producing the syllable sequence in this way is costlier than producing them with smaller frequency difference. Accordingly, it can also be at least partly responsible for individual differences, as we can assume that only individuals in good condition can produce sequences with large frequency differences between the consecutive syllables. In a previous study, we found that collared flycatcher females have a preference for individual song content, but we were not able to show evidence for the existence of “sexy syllables” (Garamszegi et al. 2012). One explanation could be that not the occurrence of some single syllable types but syllable types in a special order are important in female choice.

It is also possible that memorization constraints influence on how syllables are ordered, and particular sequences are promoted to be learnt and produced. For instance, in the zebra finch (*Taeniopygia guttata*), the sequences of syllables are transmitted socially and used stereotypically (Bruno and Tchernichovski 2019). Motifs have been described in the song of the closely related pied flycatcher (Espmark and Lampe 1993), in which syllable types are socially learnt (Eriksen et al. 2009, 2011); however, we have no information if sequence learning takes place in the old-world flycatchers so far. In this study, we also showed that motifs with up to five syllables commonly occur in collared flycatcher, which can largely determine the sequential organization. While we found significant correlation between the number of motif types and average shortest path, the explained variance was small. Therefore, while the higher-order relationships can be detected with the network method based on the first-order relationships, further studies are needed to clarify the existing higher-order patterns and their relationships with individual traits by using non-Markovian approaches (Kershenbaum et al. 2014).

It is also plausible that females prefer males that sing locally frequent syllable sequences that are specific to the population. Populations can differ in the sequential organization of songs, as it has been shown in the common chaffinch (*Fringilla coelebs*, Lachlan et al. 2013). While female preference for local song content was shown before in other species (Baker et al. 1987; Danner et al. 2011; Mortega et al. 2014), the importance of sequential organization related to female preference was not studied yet. It can be especially interesting to study this phenomenon in species where the syllables are learnt, and the sequences can be changed throughout life based on the males’ experiences.

### Consistency of sequential organization

We found repeatability estimates that cover a range between 0.43 and 0.49 for the average degree in the within-day and between-day contexts and in small-worldness within days. This magnitude is similar to what has been found for the repeatability of behavior in general (Bell et al. 2009) and for other song traits in particular (Forstmeier et al. 2002; Garamszegi, Hegyi, et al. 2006; Ren and An 2007; Zsebők et al. 2017). These results suggest that the ordering of syllables consistently differs among individuals at least between days in the breeding season and may be under natural or sexual selection. In the between-year scale, we found small repeatability estimates that cannot be statistically differentiated from zero for any of the studied network variables, suggesting that consistent among-individual differences disappear over longer time scales, for example, because of aging or experience (see also the next session).

Despite the importance of repeatability calculations from the statistical and the biological perspectives in terms of the accuracy of measurements and the function of traits in the communication, respectively (Zsebők et al. 2017), according to our knowledge, previous studies have not provided estimates of repeatability for sequential organization. However, an extreme example for the coding potential of sequential organization was shown in the Cassin’s vireo (Arriaga et al. 2016), where individuals had unique sequences implying high repeatability.

### Relationships with male quality and fitness

Among the phenotypic traits investigated, only age appeared to be an important determinant of the sequential organization of syllables. In particular, older males had a smaller average degree than young ones indicating that older individuals tended to produce syllable sequences with more unique syllables (not used in other sequences) compared to younger males. In other words, the sequences of older individuals are less similar to random sequences than sequences of younger individuals; therefore, older individuals have larger orderliness in their sequences. Such age effects were also reflected by the low repeatability of this network variable in the between-year context. Importantly, using repeated recordings from the same individuals, we found that the observed among-individual patterns were at least partly shaped by within-individual age-related changes. Accordingly, some earlier studies including the sister species, pied flycatcher, have reported that birds may change their motifs or song types in their lifetime, which phenomenon can partially explain the age effects we detected here (Espmark and Lampe 1993; Nordby et al. 2000; Woolley and Rubel 2002; Kiefer et al. 2014). Accordingly, similar results were found at song sequence levels in common nightingales (Weiss et al. 2014), where older males sang with larger average shortest path and larger small-worldness. In any case, syllable sequences may have a potential to function as a reliable signal of male age that is apparently an important trait related to male quality in collared flycatcher as older males have higher pairing success than younger individuals (Szász et al. 2019).

The year-to-year within-individual changes of syllable sequential organization may indicate the effect of a long-term learning process. It has been already shown that learning the rules of sequential ordering and learning particular syllable types require different cognitive mechanisms (Chaiken and Bohner 2007; Lipkind et al. 2018). Our earlier findings indicated that repertoire size may increase with the age in collared flycatchers (Garamszegi et al. 2007), and the current analysis suggests that the open-ended learning process is involved in the shaping of sequential organization of syllables independent of the processes of acquiring the particular elements of the repertoire.

We observed no relationships between body condition or arrival date and any network variables we focused on. One explanation for the lack of these relationships could be that the sequential organization is shaped by a long-term learning process and is largely independent of immediate food availability and other environmental conditions that affect immediate body condition and arrival date of males. However, we cannot exclude that some other uncharacterized aspects of the momentary components of condition, such as health status or physiological stress (Garamszegi et al. 2004; Garamszegi, Merino, et al. 2006), might have an effect on the sequential organization of syllables. Furthermore, although we recorded the songs in the field according to a strict protocol, the possibility that the males were in different environmental conditions cannot be ruled out. For example, in common nightingales, the sequential organization of songs depends on the order of the neighbors’ songs (Weiss et al. 2014); in rock hyraxes (*Procavia capensis*), the age, residency, and tenure have a combined effect on the ordering of their vocal signals (Demartsev et al. 2019); also, banded wrens (*Thryothorus pleurostictus*) use different song type-switching patterns according to the territorial situation (Trillo and Vehrencamp 2005). Therefore, in our study, the context specificity of sequential organization may have generated large and uncontrolled environmental variability in network variables that may have blurred the relationships with male traits.

We found no relationships between the fitness-related variables (pairing success and survival to the next year) and the network variables. Further studies are crucial to reveal the function of sequential organization of syllables in the intersexual selection related to polygyny and extrapair copulations that we did not study here but occur in our studied population (Rosivall et al. 2009; Herényi et al. 2014). Furthermore, we must emphasize that intrasexual selection may also shape the sequential organization of syllables, as sequential characteristics can be function in territorial interactions (Trillo and Vehrencamp 2005; Weiss et al. 2014; Demartsev et al. 2019).

## CONCLUSIONS

We presented here a sequential analysis of bird song on a large number of individuals to reveal consistent individual differences in syllable sequence organization in birdsong by using network analysis. We showed that the nonrandom ordering of syllables within the songs of the collared flycatcher can potentially evolve as a reliable signal. The organization of syllable sequences provides information about the singer, as it shows patterns that are consistent within individuals, at least in between-day time scale in the breeding season and correlates with male age. We also showed that the acoustic characteristics and the usage of higher-order motifs are related to the sequential organization. To directly demonstrate how the sequential characteristics of songs function in the intra- or intersexual communication pathways further studies are needed.

## Supplementary Material

araa104_suppl_Supplementary-Figure-S1

araa104_suppl_Supplementary-Figure-S2

araa104_suppl_Supplementary-Material
