# Supplementary figures and images for: Sequential organization of birdsong: relationships with individual quality and fitness
Source: Behav Ecol. 2020 Oct 30;32(1):82–93. doi: 10.1093/beheco/araa104 (PMC7937035; doi:10.1093/beheco/araa104)

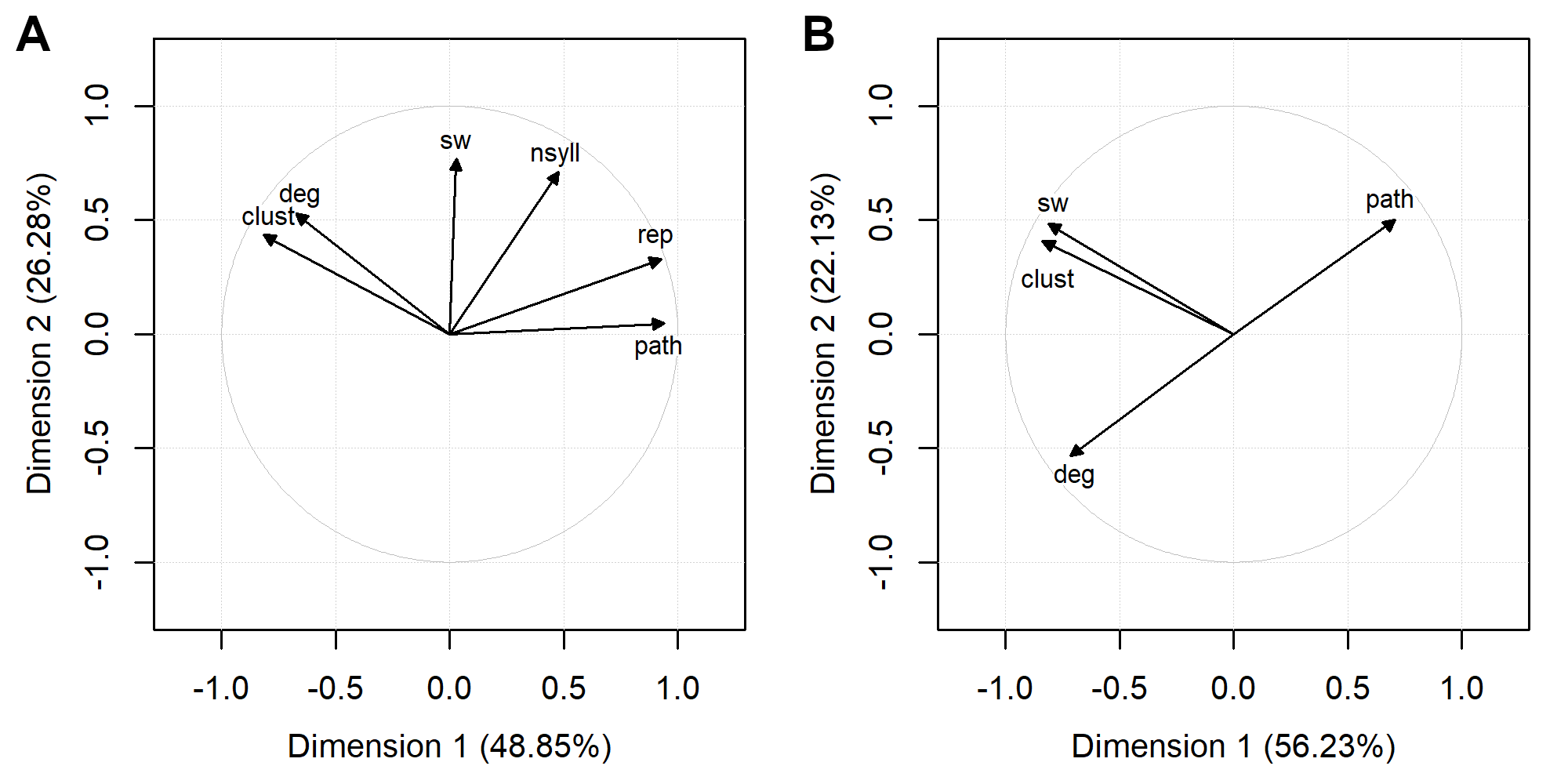

Supplement: araa104_suppl_Supplementary-Figure-S1 [file araa104_suppl_supplementary-figure-s1.png]

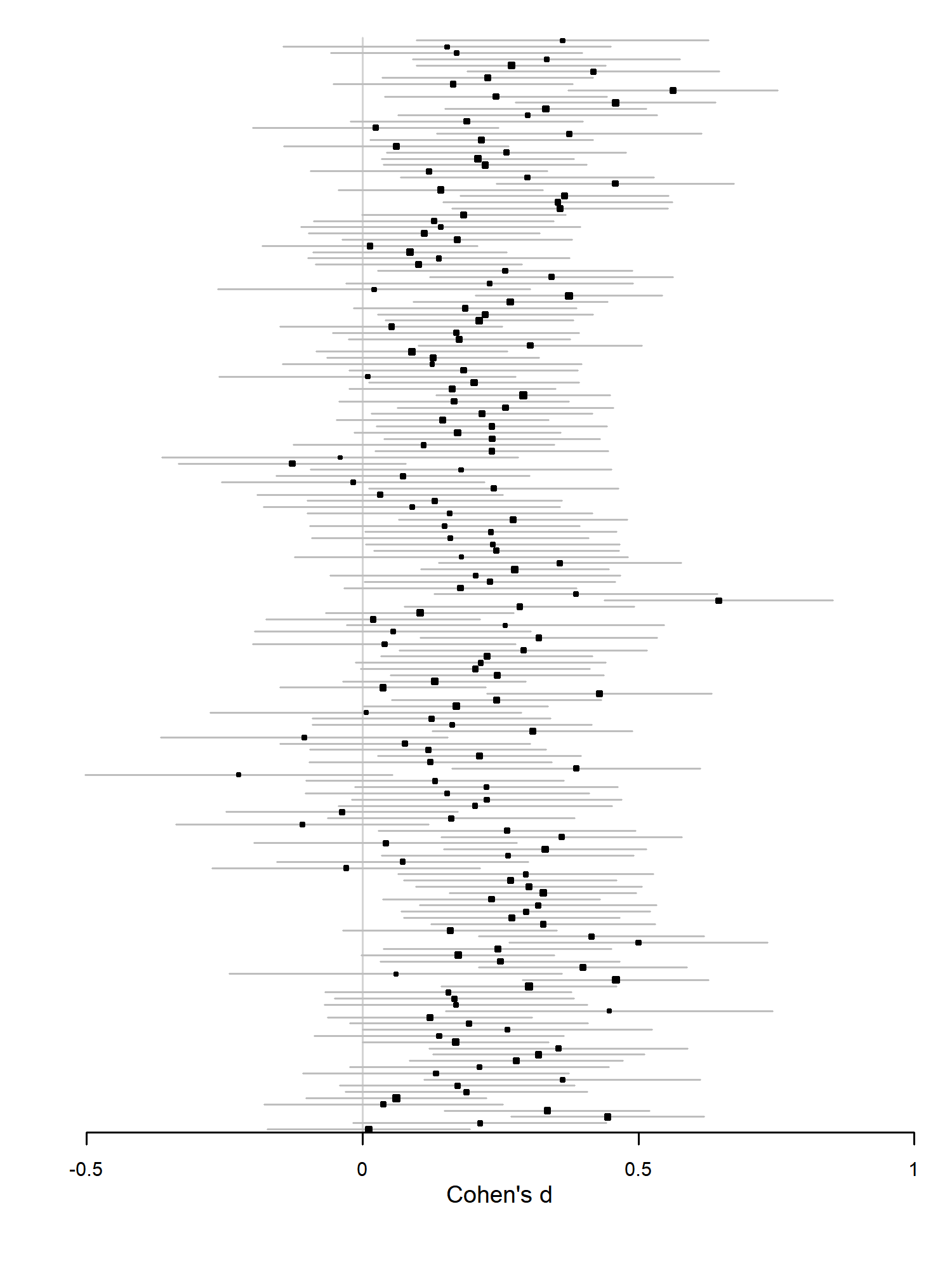

Supplement: araa104_suppl_Supplementary-Figure-S2 [file araa104_suppl_supplementary-figure-s2.png]
