# Supplementary material for: Sequential organization of birdsong: relationships with individual quality and fitness
Source: Behav Ecol. 2020 Oct 30;32(1):82–93. doi: 10.1093/beheco/araa104 (PMC7937035; doi:10.1093/beheco/araa104)
Supplement: araa104_suppl_Supplementary-Material [file araa104_suppl_supplementary-material.docx]

**Supplementary Material**

**Table S1.** Results of the PCA applied for the network variables before correction for repertoire size and number of syllables: eigenvalues and percentage of variances (N = 176).

| **Principal component** | **Eigenvalue** | **Percentage of variance** | **Cumulative percentage of variance** |
| --- | --- | --- | --- |
| PC1 | 2.93 | 48.8 | 48.8 |
| PC2 | 1.58 | 26.3 | 75.1 |
| PC3 | 0.92 | 15.3 | 90.4 |
| PC4 | 0.31 | 5.1 | 95.5 |
| PC5 | 0.20 | 3.4 | 98.9 |
| PC6 | 0.07 | 1.1 | 100.0 |

**Table S2.** Results of the PCA applied for the network variables before correction for repertoire size and number of syllables: contribution of variables in percent (N = 176).

|  | **PC1** | **PC2** | **PC3** | **PC4** | **PC5** | **PC6** |
| --- | --- | --- | --- | --- | --- | --- |
| **Repertoire size** | 27.9 | 6.5 | 0.4 | 0.3 | 23.7 | 41.1 |
| **Number of syllables** | 7.3 | 30.2 | 25.5 | 0.9 | 35.7 | 0.3 |
| **Average degree** | 14.6 | 16.7 | 25.0 | 0.1 | 36.8 | 6.7 |
| **Average shortest path** | 28.6 | 0.1 | 0.0 | 45.1 | 1.8 | 24.4 |
| **Clustering coefficient** | 21.5 | 11.3 | 4.1 | 45.5 | 2.0 | 15.7 |
| **Small-worldness** | 0.0 | 35.2 | 44.9 | 8.1 | 0.0 | 11.8 |

**Table S3.** Results of the PCA applied for the network variables after correction for repertoire size and number of syllables: eigenvalues and percentage of variances (N = 176).

| **Principal component** | **Eigenvalue** | **Percentage of variance** | **Cumulative percentage of variance** |
| --- | --- | --- | --- |
| PC1 | 2.2 | 56.2 | 56.2 |
| PC2 | 0.9 | 22.1 | 78.4 |
| PC3 | 0.7 | 17.1 | 95.5 |
| PC4 | 0.2 | 4.5 | 100.0 |

**Table S4.** Results of the PCA applied for the network variables after correction for repertoire size and number of syllables: contribution of variables in percent (N = 176).

|  | **PC1** | **PC2** | **PC3** | **PC4** |
| --- | --- | --- | --- | --- |
| **Average degree** | 21.6 | 30.6 | 31.3 | 16.6 |
| **Average shortest path** | 21.0 | 26.6 | 38.9 | 13.4 |
| **Clustering coefficient** | 29.6 | 17.8 | 16.3 | 36.3 |
| **Small-worldness** | 27.8 | 25.0 | 13.4 | 33.8 |

**Table S5.** Results of meta-analysis for the comparison of network variables obtained for original and randomized sequences (N = 176).

| **Network variable** | **Mean** | **Lower CI** | **Upper CI** |
| --- | --- | --- | --- |
| average degree | -13.5 | -14.2 | -12.8 |
| average shortest path | 12.6 | 11.2 | 14.0 |
| clustering coefficient | -5.8 | -6.2 | -5.4 |
| small-worldness | -1.9 | -2.3 | -1.6 |

**Table S6.** Repeatabilities of the network variables on three different time-scales (within-day: N = 34; between-day: N = 24, between-year: N = 13).

| **Parameter** | **Time-scale** | **Mean** | **Lower CI** | **Upper CI** |
| --- | --- | --- | --- | --- |
| average degree | within day | 0.43 | 0.11 | 0.67 |
|  | between days | 0.44 | 0.05 | 0.71 |
|  | between years | 0.04 | 0.00 | 0.56 |
| average shortest path | within day | 0.31 | 0.00 | 0.56 |
|  | between days | 0.32 | 0.00 | 0.64 |
|  | between years | 0.00 | 0.00 | 0.51 |
| clustering coefficient | within day | 0.14 | 0.00 | 0.47 |
|  | between days | 0.28 | 0.00 | 0.62 |
|  | between years | 0.00 | 0.00 | 0.55 |
| small-worldness | within day | 0.49 | 0.19 | 0.71 |
|  | between days | 0.31 | 0.00 | 0.63 |
|  | between years | 0.17 | 0.00 | 0.67 |

**Figure S1.** Relationships of the variables in PCA space before and after controlling for the effects of repertoire size and number of syllables for directed network. The axes are the first two principal components. The more the arrows point to the same direction, the more similar those corresponding variables are (rep: repertoire size, nsyll: number of syllables, degree: average degree, path: average shortest path, clust: clustering coefficient, sw: small-worldness).

**Figure S2.** Forest plot for the meta-analysis of the frequency differences of consecutive syllables between original and randomized sequences (N = 176). For the underlying statistics, see Results in the main text.

**Figure S1.**

**
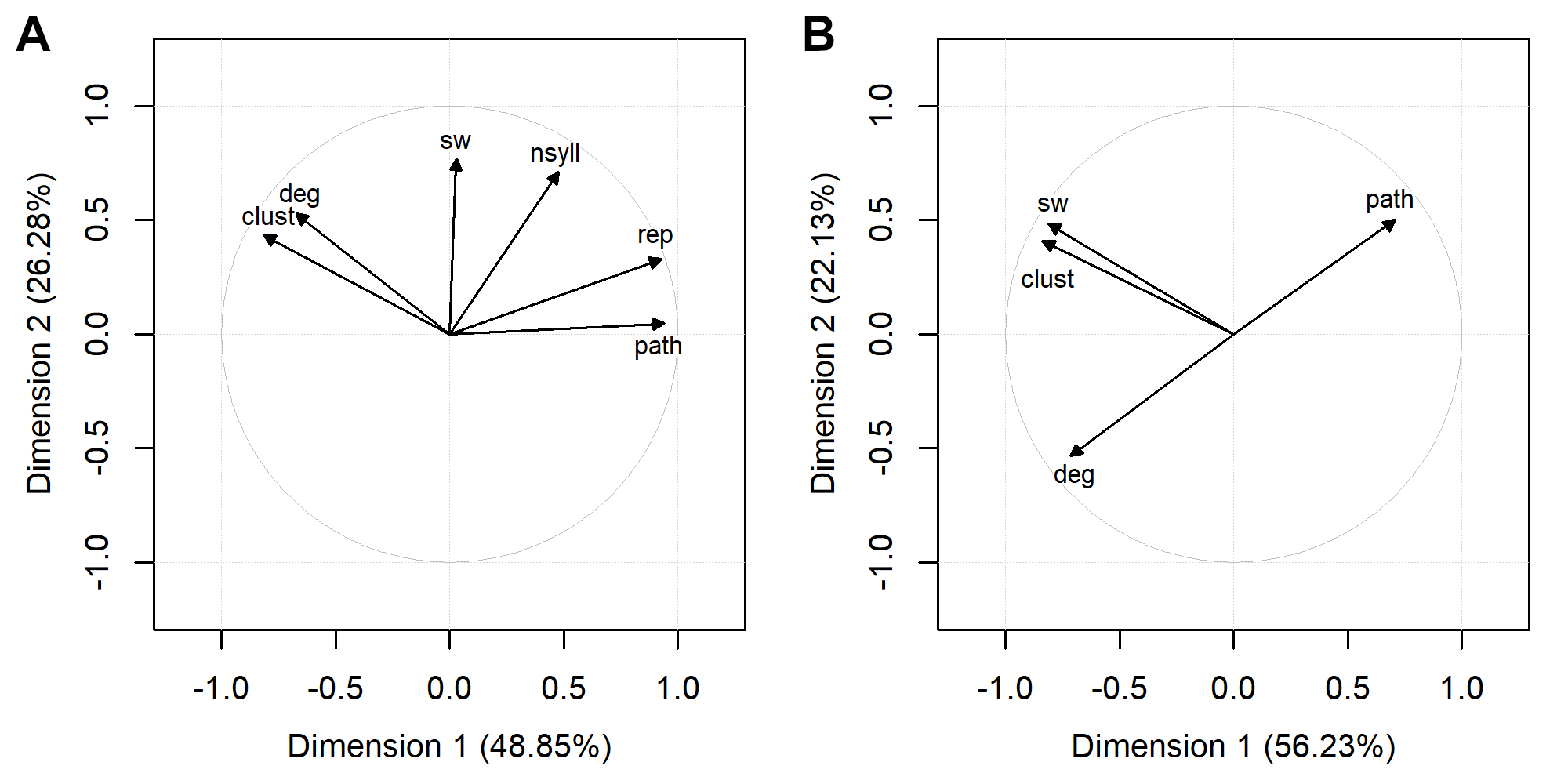
**

**Figure S2.**

**
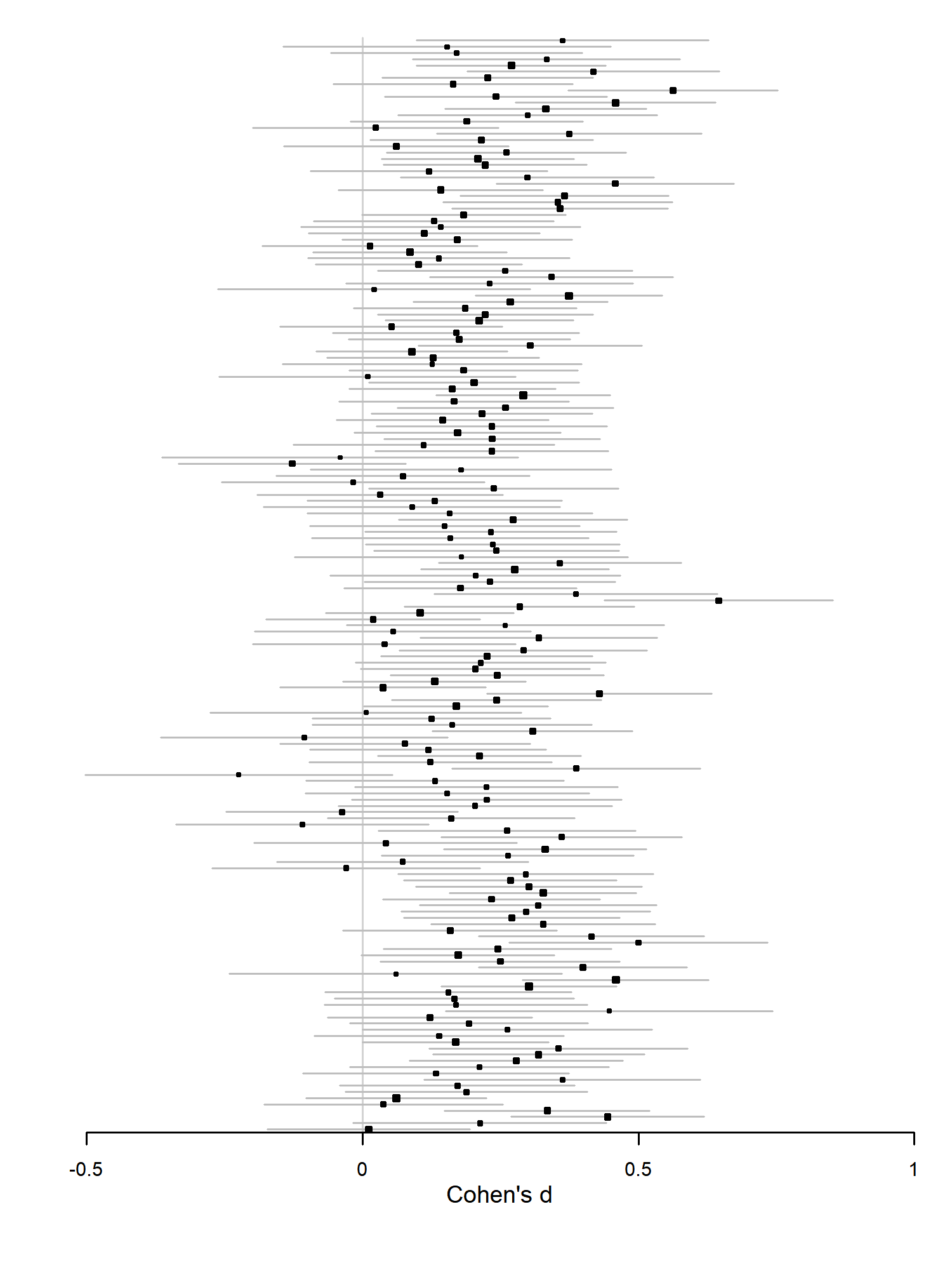
**
